# Gray matter density loss in essential tremor: a lobule by lobule analysis of the cerebellum

**DOI:** 10.1186/s40673-017-0069-3

**Published:** 2017-07-03

**Authors:** Jonathan P. Dyke, Eric Cameron, Nora Hernandez, Ulrike Dydak, Elan D. Louis

**Affiliations:** 1 Department of Radiology, Citigroup Biomedical Imaging Center, Weill Cornell Medicine, New York, NY USA; 2 School of Health Sciences, Purdue University, West Lafayette, IN USA; 3 Department of Radiology and Imaging Sciences, Indiana University School of Medicine, Indianapolis, IN USA; 4 Department of Neurology, Yale School of Medicine, Yale University, New Haven, CT USA; 5 Department of Chronic Disease Epidemiology, Yale School of Public Health, Yale University, New Haven, CT USA; 6 Center for Neuroepidemiology and Clinical Neurological Research, Yale School of Medicine, Yale University, New Haven, CT USA; 7 Departments of Neurology and Chronic Disease Epidemiology Yale School of Medicine, Yale School of Public Health Yale University, New Haven, CT USA

**Keywords:** Essential tremor, MRI, Cerebellum, Volumetrics, Lobes, Degeneration

## Abstract

**Background:**

The pathophysiological basis for essential tremor (ET) remains unclear, although evidence increasingly links it to a disordered and perhaps degenerative cerebellum. Prior imaging studies have treated the cerebellum *en bloc.* Our hypothesis was that regional differences in cerebellar gray matter (GM) density may better distinguish ET cases from controls.

Forty-seven ET cases and 36 control subjects were imaged using magnetic resonance imaging (MRI). The cerebellum was segmented into 34 lobes using a Spatially Unbiased Infra-Tentorial Template (SUIT) atlas within the Statistical Parametric Mapping (SPM) analysis package. Age, gender and Montreal Cognitive Assessment (MoCA) scores were regressed out from the statistical models to isolate group effects. ET cases were further stratified into phenotypically-defined subgroups. The Benjamini-Hochberg False Discovery Rate procedure (BH FDR) (α = 0.1) was used to correct for multiple comparisons.

**Results:**

When all ET cases and controls were compared, none of the regions met the BH FDR criteria for significance. When compared with controls, ET cases with head or jaw tremor (*n* = 27) had significant changes in GM density in nine cerebellar lobules, with a majority in the left cerebellar region, and each meeting the BH FDR criteria. Likewise, ET cases with voice tremor (*n* = 22) exhibited significant changes in 11 lobules in both left and right regions and the vermis. These analyses, in sum, indicated decreases in GM density in lobules I-IV, V, VI, VII and VIII as well as the vermis. ET cases with severe tremor (*n* = 20) did not show regions of change that survived the BH FDR procedure when compared to controls.

**Conclusions:**

We showed that ET cases with various forms of cranial tremor differed from controls with respect to cerebellar GM density, with evidence of GM reduction across multiple cerebellar regions. Additional work, using a lobule-by-lobule approach, is needed to confirm these results and precisely map the regional differences in ET cases, subgroups of ET cases, and controls.

## Background

Essential tremor (ET) is a neurological disease whose chief clinical feature is kinetic tremor of the arms; it is the most common tremor disorder in humans [1, 2]. The pathophysiological basis for ET is still under active investigation, although evidence increasingly links it to a disordered and perhaps degenerative cerebellum [3, 4].

Multi-modality neuroimaging studies have used Magnetic Resonance Imaging (MRI), Positron Emission Tomography (PET) and Single Photon Emission Computed Tomography (SPECT) to study structural and metabolic changes in ET [5, 6]. These studies have reported metabolic and structural changes in the cerebellum [7–9]. However, they have yielded conflicting results with regards to generalized cerebellar atrophy. Hence, further study is needed.

Voxel based morphometry (VBM) is frequently used to identify clusters of voxels with differing percentage gray matter (%GM) density. These clusters are determined through statistical analysis and frequently overlap specific anatomical boundaries of individual cerebellar lobules. Our study accurately identifies contributions of %GM density change within specific cerebellar lobules of interest by using a Spatially Unbiased Infra-tentorial Template (SUIT) of the cerebellum and brainstem [10]. This atlas was normalized on a subject specific basis to the GM segmented data and the %GM density was quantified in each cerebellar lobule. The %GM density is assumed to be a close proxy of brain atrophy and has been used to quantitate the degree of neurodegeneration in Alzheimer’s and Parkinson’s disease [11, 12]. We postulate that our lobule by lobule approach may better distinguish changes in %GM density in subjects with ET from controls or subtypes of ET from controls.

Our study also included a detailed clinical evaluation of ET patients, which allowed us to assess multiple phenotypic features. These included the severity of action tremor (total tremor score, TTS), the presence of head (i.e., neck) or jaw tremor, and the presence of voice tremor. As cognitive dysfunction has been associated with ET as well, we administered the Montreal Cognitive Assessment (MoCA) score, a measure of global cognitive function. Detailed phenotyping is important as there is a sense that ET may be a family of diseases rather than a unitary entity, and etiological and pathophysiological features could track with certain phenotypes [13].

Aside from possible diagnostic value, the current technique has the potential to be applied to serial studies to assess disease progression as a function of time. Knowledge of specific cerebellar lobule degeneration may also be used to localize pathways and models of disease within the cerebellum as linked to tremor.

## Methods

### Subjects and clinical evaluation

Forty-seven ET cases (24 M/23F, age 76.0 ± 6.8 years, TTS 20.3 ± 6.2, MoCA score 27.5 ± 2.2) and 36 normal control subjects (10 M/26F, age 73.2 ± 6.7 years, TTS 5.3 ± 2.5, MoCA score 28.1 ± 1.7) were recruited for this study. The average age of onset of ET was 40.3 ± 21.5 years with a range from 5 to 75 years, and 5 ET subjects having an age of onset ≥65 years. This sample size was well within the range of that of prior volumetric studies of ET (e.g., 10 ET cases and 13 controls [14], 19 ET and 19 controls [15], 39 ET cases and 36 controls [7]). The study protocol was reviewed and approved by the Yale, Cornell and Purdue Human Subjects Institutional Review Boards. Written informed consent was obtained from each subject prior to participation in the study.

ET cases were recruited from a clinical-epidemiological case-control study of ET, one of the author’s (E.D.L.) neurological practices and study advertisements [16]. Inclusion criteria were a diagnosis of ET from a treating neurologist and a willingness to undergo an MRI scan. Exclusion criteria included heavy exposure to ethanol (as defined previously) [17], a history of a neurodegenerative disease (Alzheimer’s disease, Parkinson’s disease, etc.), prior deep brain stimulation or other neurosurgery or a contraindication for MRI. Normal control subjects were recruited during the same time period and from the same sources as the ET cases with some being spouses of the ET cases. They were matched to ET cases based on age. As cases were more readily available, their recruitment occurred more easily that those of controls, and this contributed to an unequal number of cases and controls. Exclusion criteria for controls included a history of ET and a family history of ET (i.e., a reportedly affected first-degree or second-degree relative).

An in-person assessment was performed by a trained research assistant. Demographic and clinical data were collected and, as cognitive deficits have been reported in ET, the Montreal Cognitive Assessment (MoCA) was performed as a brief assessment of cognitive function [18]. During the in-person assessment, a videotaped neurological examination was also performed on all cases and controls. This included one test for postural tremor and five for kinetic tremor (12 tests total) as well as sustained phonation, conversational speech and a reading aloud task. A senior neurologist specializing in movement disorders (E.D.L.) used a reliable and valid clinical rating scale, the Washington Heights-Inwood Genetic Study of Essential Tremor (WHIGET) rating scale [19], to rate postural and kinetic tremor during each test: 0, 0.5, 1, 1.5, 2 and 3, resulting in a total tremor score (range, 0–36). Head (i.e., neck) and jaw tremors were noted as present or absent on examination, and a head/jaw tremor score was assigned to each case [0 = Absent, 1 = Present in Head or Jaw, 2 = Present in Head and Jaw]. In addition, voice tremors were noted as present or absent on examination. Diagnoses of ET were re-confirmed by the neurologist (E.D.L.) based on the history and videotaped neurological examination. WHIGET diagnostic criteria were applied (moderate or greater amplitude kinetic tremor [tremor rating ≥ 2]) during three or more tests or the presence of a head tremor, in the absence of Parkinson’s disease, dystonia or another cause.

### MRI acquisition

MRI exams were performed on a 3.0 Tesla Siemens Tim Trio scanner (Siemens Healthcare, Erlangen, Germany), equipped with a 32-channel head coil. All scans were performed at Weill Cornell Medicine at the Citigroup Biomedical Imaging Center. In order to determine voxel tissue composition and account for partial volume components, high-resolution MPRAGE images were acquired (TR/TE/TI = 2300/2.91/900 ms, flip angle = 9°, bandwidth: 240 Hz/pixel, voxel size: 1.0 mm × 1.0 mm × 1.2 mm, GRAPPA = 2).

### Data processing and analysis

**Fig. 1:**
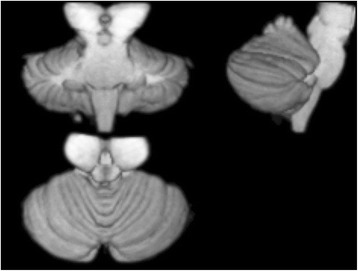
A 3D representation of the segmented brainstem and cerebellum produced by SUIT

MRI data processing was performed using SPM12 (University College London, Wellcome Trust Centre for Neuroimaging, UK) using the SUIT toolbox [20]. This refers to the Spatially Unbiased Infra-tentorial Template of the cerebellum and brainstem [21]. SPM12 was specifically chosen as previous versions (SPM5, SPM8) were noted to stretch the cerebellum in the z-direction during the normalization procedure. The first step was to “isolate” the cerebellum and brainstem using the SUIT toolbox on the high resolution T_1_-Weighted MPRAGE images (Fig. 1). Gray matter segmentation was performed within SPM using a modified Gaussian mixture model. This model segments GM voxels based on likelihood and prior probability maps compiled from a large sample set.

The next step was to normalize or deform each cerebellum and brainstem into the SUIT atlas template. This procedure aligns individual fissures and reduces their spatial spread as well as improves on alignment of the deep cerebellar nuclei. The atlas contains 34 lobes in the left, right and vermal regions [22]. The following 13 regions were defined in the left and right cerebellum, respectively: Crus I, Crus II, Dentate, Fastigial, Interposed, I-IV, V, VI, VIIb, VIIIa, VIIIb, IX, X. The following 8 regions were defined in the vermis: Crus I, Crus II, VI, VIIb, VIIIa, VIIIb, IX, X. Once the normalization matrix was determined, the final step inverted the transformation and resliced the SUIT cerebellar atlas back into subject space. This produced a subject specific cerebellar atlas with the number of voxels in each lobule converted back to a volume in cc knowing the voxel size of 1.0 mm × 1.0 mm × 1.2 mm.

**Fig. 2:**
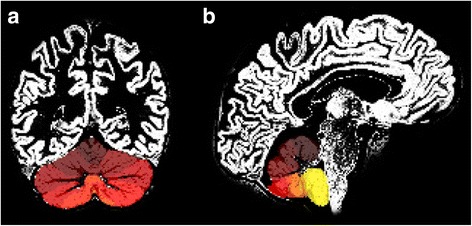
Overlay of the resliced subject specific SUIT template onto the GM segmented whole brain image. [**a** Coronal plane, (**b**) Sagittal plane]

The volume of each lobule in the atlas contained contributions from GM, white matter (WM) and cerebrospinal fluid (CSF). Any changes in GM density caused by cerebellar atrophy might be masked by lesser changes in absolute lobular volume which may occur with normal aging. The GM segmented map was resliced in SPM to match the matrix size of the subject specific SUIT atlas (Fig. 2). Code was written in-house (J.P.D.) using IDL 8.1 (Exelis Visual, Broomfield,CO) to mask each lobule in the SUIT atlas and multiply by the GM segmented image. The sum of all voxel values in the resulting image product represented the total GM volume within each lobule. A final quantitative ratio of GM volume/total lobular volume yielded the exact %GM density in each lobule. This ratio was independent of the size of the lobule and could be compared across groups.

### Statistical analyses

All analyses were performed in R (version 3.3.2). We used Student’s t tests to compare the features of ET cases vs. control subjects. Linear regression models were performed with the %GM density from each of the 34 cerebellar regions as the outcome, with group (control vs. ET), age, gender and MOCA score as independent predictors. The Benjamini-Hochberg method was used to calculate the False Discovery Rate (FDR) and correct for multiple comparisons [23, 24]. Statistical tests resulting in *p* < 0.05 were considered significant with an FDR cutoff of (α = 0.1). In additional models, we compared three phenotypically-defined subgroups of ET cases to controls (i.e., ET cases with head or jaw tremor (HJT), ET cases with voice tremor (VT), and ET cases with severe arm tremor defined by the upper quartile of TTS [≥23]).

## Results

ET cases and controls did not differ significantly with respect to age (*p* = 0.062) or MOCA score (*p* = 0.116) (Table 1). There was a slight difference with respect to gender (*p* = 0.03) in the control group, which was accounted for in the regression model. In these comparisons, age, gender, MoCA score and group were incorporated as continuous and discrete independent variables and regressed against the regional %GM density of the 34 cerebellar regions.

**Table 1:** Demographic data on the normal control and ET cases

	Controls (*n* = 36)		ET (*n* = 47)			
	Mean	Stdev	Mean	Stdev	t	p
Age (years)	73.1	6.7	76.0	6.8	−1.90	0.062
Gender	10 M(28%) 26F(72%)		24 M(51%) 23F(49%)		2.20	0.030
MoCA	28.1	1.7	27.4	2.5	1.59	0.116
Age of Onset (years)	NA	NA	41.0	20.7	NA	NA
HJT score	0.0	0.0	1.0	1.1	NA	NA
VT score	0.0	0.0	0.5	0.5	NA	NA
TTS	5.3	2.5	20.4	6.2	−15.21	<0.0001

The total tremor score (TTS) has a range of 0 to 36. The head/jaw tremor score (HJT) ranges from 0 to 2. The voice tremor score (VT) is binary (0 or 1). *MoCA* Montreal Cognitive Assessment, *NA* not applicable

**Fig. 3:**
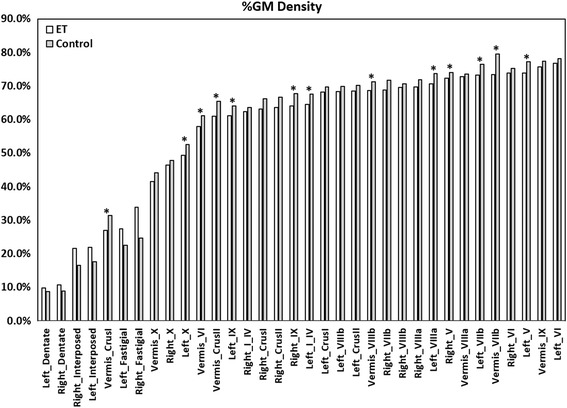
A plot showing the %GM density within 34 cerebellar regions highlighting regions of significant decrease in ET with HJT (*n* = 27) and VT (*n* = 22) versus controls with an *asterisk*

**Fig. 4:**
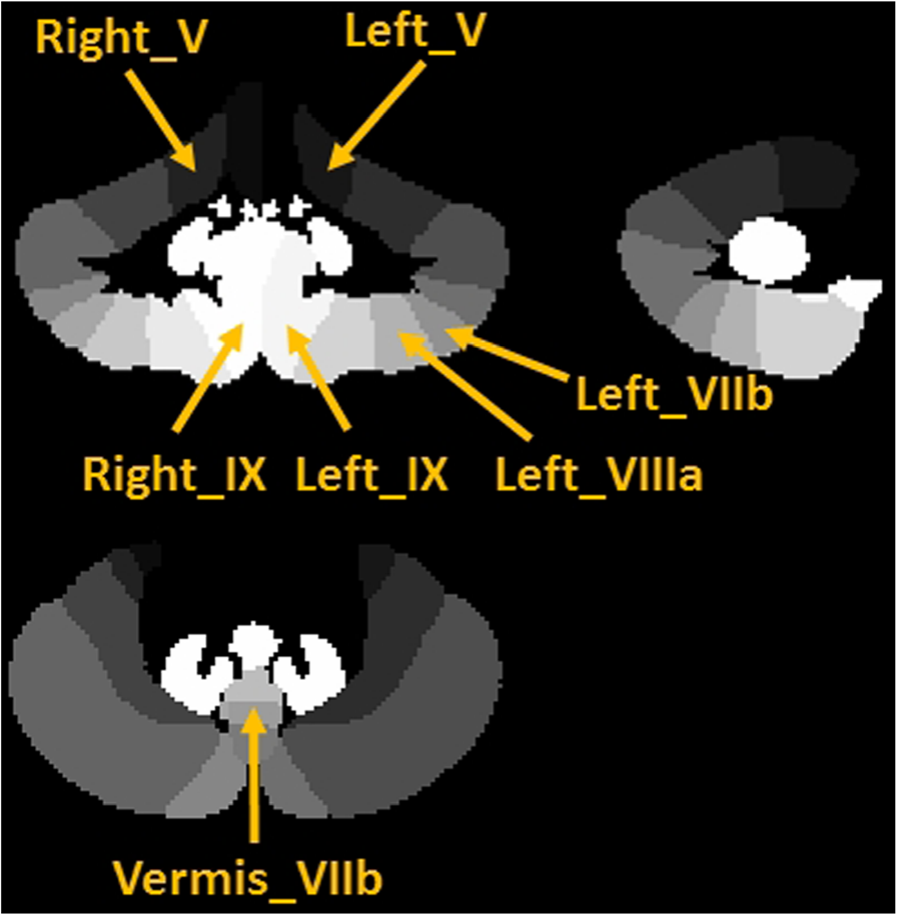
A cerebellar atlas from SUIT is shown labelled with representative regions that showed a significant loss in %GM between control subjects and subgroups of ET subjects. [Note that additional regions of significance could not be displayed given they are visible on different images]

Comparing the %GM density in all ET vs. control subjects resulted in no significant differences after correction for multiple comparisons. In additional models, we compared three phenotypically-defined subgroups of ET cases to controls. When compared with controls, ET cases with head or jaw tremor (HJT; *n* = 27) showed significant decreases in %GM density that met the BH FDR criterion in the following regions, presented in order of significance: Right_IX (*p* = 0.001), Left_V (*p* = 0.004), Left_VIIIa (*p* = 0.009), Left_IX (*p* = 0.010), Vermis_VIIb (*p* = 0.011), Left_VIIb (*p* = 0.013), Left_X (*p* = 0.014), Left_I_IV (*p* = 0.018) and Right_V (*p* = 0.021) (Table 2). Compared to controls, ET cases with voice tremor (VT; *n* = 22) exhibited significant decreases in %GM density that met the BH FDR criterion in the following regions, presented in order of significance: Right_IX (*p* = 0.001), Vermis_VIIb (*p* = 0.004), Left_IX (*p* = 0.005), Left_V (*p* = 0.006), Left_X (*p* = 0.008), Vermis_CrusII (*p* = 0.012), Vermis_CrusI (*p* = 0.016), Vermis_VI (*p* = 0.019), Left_I_IV (*p* = 0.025), Vermis_VIIIb (*p* = 0.026) and Right_V (*p* = 0.026) (Table 2). Lastly, ET cases with severe tremor (TTS ≥ 23; *n* = 20) showed no significant decreases compared to controls after correcting for multiple comparisons. The average %GM density for each region comparing all ET (*n* = 47) and control subjects (*n* = 36) is plotted in Fig. 3; the asterisks label significant regions of decreased %GM in ET with HJT (*n* = 27) and VT (*n* = 22) versus controls (Table 2). Figure 4 labels representative lobules of significant decrease between control subjects and subgroups of ET subjects.

**Table 2:** Comparison of lobular %GM density

	ET w/HJT vs. Controls	ET w/VT vs. Controls
	*n* = 27 vs *n* = 36	*n* = 22 vs *n* = 36
Left_I-IV	(*p* = 0.018)	(*p* = 0.025)
Left_V	(*p* = 0.004)	(*p* = 0.005)
Left_VIIb	(*p* = 0.013)	
Left_VIIIa	(*p* = 0.009)	
Left_IX	(*p* = 0.010)	(*p* = 0.005)
Left_X	(*p* = 0.014)	(*p* = 0.008)
Right_V	(*p* = 0.021)	(*p* = 0.026)
Right_IX	(*p* = 0.001)	(*p* = 0.001)
Vermis_CrusI		(*p* = 0.016)
Vermis_CrusII		(*p* = 0.012)
Vermis_VI		(*p* = 0.019)
Vermis_VIIb	(*p* = 0.011)	(*p* = 0.004)
Vermis_VIIIb		(*p* = 0.026)

Regions of significance are shown (*p* < 0.05) that met the BH FDR criterion. Abbreviations [*HJT* Head/Jaw Tremor (i.e., presence of head or jaw tremor on examination), *VT* Voice Tremor (i.e., presence of voice tremor on examination)]. In these comparisons, age, gender, MOCA score and group were incorporated as continuous and discrete independent variables and regressed against the regional %GM density of the 34 cerebellar regions

## Discussion

In this study we showed that ET cases with various forms of cranial tremor differed from controls with respect to cerebellar GM, with evidence of GM reduction across several cerebellar regions. Our study is unique in the sense that it quantified %GM density in 34 distinct lobules of the cerebellum in cases and age-matched controls using high resolution 3D T_1_-Weighted MRI. We are aware of one other study that used a similar but not identical approach, namely, a voxel based morphometry study of 28 manually defined lobules of the cerebellum in 39 ET cases and 36 controls based on a cerebellar atlas from a single subject. In that study, a reduction in GM volume was noted in vermis lobules VI and VII, which are contiguous areas of the cerebellar vermis [7]. All other studies have treated the cerebellum as an entire organ, as two hemispheres or as anterior and posterior regions, but have not used a lobule by lobule approach [14, 25]. One partial exception is a study that examined 6 regions of the cerebellum (bilateral dentate and bilateral cerebellar lobules VI and VIII), noting that there was GM loss in lobule VIII [15].

Voxel based morphometry studies identify clusters of change between two groups but do not specifically delineate boundaries of individual cerebellar lobules. Overlap of these clusters across lobular boundaries and contribution from each lobule is uncertain. Physiologically, cerebellar function may cross these lobular boundaries to include multiple lobules as shown in several fMRI studies in ET [5, 26]. Typically, only the center of a statistically significant cluster in MNI or Talaraich space is cross-referenced to a location within a published cerebellar atlas taken from a single subject [27]. A source of error in the resulting cerebellar lobule boundaries may also occur when non-linear normalization forces unique subject specific cerebellar architecture into a fixed template. Additionally, automated segmentation techniques such as FreeSurfer do not identify specific lobular boundaries of the cerebellum. Our study specifically utilizes a lobular probabilistic atlas of the cerebellum based on 20 subjects to identify regional %GM density change between subgroups.

In the current study, we detected a reduction in %GM density in ET cases, and more specifically, those with head and jaw tremor and voice tremor, across a range of cerebellar lobules. These changes in GM density could not be accounted for by variations in age, gender or MoCA score. We hypothesize that a decrease in %GM density between groups would represent atrophy in a specific cerebellar lobule. However, a decrease in %GM could also be accounted for by an increase in %WM [5]. Only one study found changes in both cerebellar GM and WM density in ET using a VBM analysis uncorrected for multiple comparisons [28].

One prior study examined 6 regions of the cerebellum (bilateral dentate and bilateral cerebellar lobules VI and VIII) and reported GM loss in lobule VIII among 19 ET cases vs. 19 controls [15]. The same authors, using functional MRI in 21 ET cases and 21 controls, performed a lobule by lobule analysis of the cerebellum, reporting tremor-related activations bilaterally in the cerebellum in left and right lobules V and VI and in right lobules VIIIa and VIIIb [29]. As noted by the authors, lobules V and VI have strong primary somatosensory representation and lobule VIIIb, a secondary representation. Our study confirmed decreases in %GM density in lobules V and VIII as well as additional regions found to be different in prior imaging studies of ET [7, 16, 19]. We also report decreases in GM density in lobules I-IV, V, VI, VII and VIII as well as the vermis (Table 2). Additional work, using a lobule-by-lobule approach, is needed to confirm these results and precisely map the regional differences in ET cases, subgroups of ET cases, and controls.

Based on postmortem studies, a number of changes have been described in the ET cerebellum. Some though not all studies have noted the presence of Purkinje cell loss.Similarly, marked reductions in the Purkinje cell dendritic arbor have been noted in ET cases compared to controls; before degeneration, the metabolic economy of neurons is severely challenged, and they are unable to maintain their extensive cytoskeleton, and this can manifest as regressive changes in dendritic morphology (e.g., a truncation of the dendritic arbor) [30]. Studies such as ours and those mentioned above suggest that such cellular changes may be accompanied by reductions in GM density in ET cases in the cerebellum.

## Conclusions

Our results show that the presence of cranial tremor and voice tremor in ET may be manifested in a reduction in lobular cerebellar %GM density between groups independent of age, gender or MOCA score. Knowledge of MRI determined regional changes in this disease may add to information on the role of the cerebellum in ET. Identification of regions with the greatest degree of %GM density change compared to that in normal aging may also aid in identifying causative mechanisms and biomarkers of disease progression.

## Abbreviations

BH-FDR: Benjamini-Hochberg False Discovery Rate
ET: Essential Tremor
GM: Gray Matter
HJT: Head-Jaw Tremor
MoCA: Montreal Cognitive Assessment Score
MRI: Magnetic Resonance Imaging
SUIT: Spatially Unbiased Infra-Tentorial Template
TTS: Total Tremor Score
VBM: Voxel Based Morphometry
VT: Voice Tremor
